# Tumor marker response to SARS‐CoV‐2 infection among patients with cancer

**DOI:** 10.1002/cam4.4646

**Published:** 2022-03-15

**Authors:** Alexander H. Gunn, Carolyn Tashie, Steven Wolf, Jesse D. Troy, Yousuf Zafar

**Affiliations:** ^1^ Duke University School of Medicine Durham North Carolina USA; ^2^ Duke Cancer Institute Durham North Carolina USA; ^3^ Department of Biostatistics and Bioinformatics Duke University Durham North Carolina USA

**Keywords:** cancer management, clinical management, clinical observations, COVID‐19, tumor markers, viral infection

## Abstract

**Background:**

Inflammatory responses from benign conditions can cause non‐cancer‐related elevations in tumor markers. The severe acute respiratory coronavirus 2 (SARS‐CoV‐2) induces a distinct viral inflammatory response, resulting in coronavirus disease 2019 (COVID‐19). Clinical data suggest carcinoembryonic antigen (CEA), carbohydrate antigen 19–9 (CA 19–9), and cancer antigen 125 (CA 125) levels might rise in patients with COVID‐19. However, available data excludes cancer patients, so little is known about the effect of COVID‐19 on tumor markers among cancer patients.

**Methods:**

We conducted a case series and identified patients with a positive SARS‐CoV‐2 PCR test, diagnosis of a solid tumor malignancy, and a CEA, CA 19–9, CA 125, or CA 27–29 laboratory test. Cancer patients with documented COVID‐19 infection and at least one pre‐ and two post‐infection tumor marker measurements were included. We abstracted the electronic health record for demographics, cancer diagnosis, treatment, evidence of cancer progression, date and severity of COVID‐19 infection, and tumor marker values.

**Results:**

Seven patients were identified with a temporary elevation of tumor marker values during the post‐COVID‐19 period. Elevation in tumor marker occurred within 56 days of COVID‐19 infection for all patients. Tumor markers subsequently decreased at the second time point in the post‐infectious period among all patients.

**Conclusion:**

We report temporary elevations of cancer tumor markers in the period surrounding COVID‐19 infection. To our knowledge this is the first report of this phenomenon in cancer patients and has implications for clinical management and future research.

## Graphical abstract

We present a case series of temporary elevations in cancer tumor markers in the period surrounding COVID‐19 infection. To our knowledge this is the first report of this phenomenon in cancer patients and has implications for clinical management and future research.

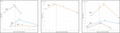

## 1 INTRODUCTION

Serial measurement of serum tumor markers—including carcinoembryonic antigen (CEA) for gastrointestinal cancers, carbohydrate antigen 19–9 (CA 19–9) for pancreatic cancer, cancer antigen 125 (CA 125) for ovarian cancer, and cancer antigen 27–29 (CA 27–29) for breast cancer—can demonstrate response to cancer treatment or signal recurrent disease. As such, the National Comprehensive Cancer Network Clinical Practice Guidelines in Oncology recommend the use of CEA for surveillance with monitoring levels every 3–6 months for the first 2 years following treatment^1^ as well as the routine use of CA 19–9, CA 125, and CA 27–29 testing.^2^, ^3^, ^4^ Elevations in tumor markers are typically interpreted as signals of non‐response to therapy or as evidence of recurrent disease, both of which can lead to alterations in clinical management and increases in patient anxiety.^5^, ^6^, ^7^, ^8^

Inflammatory responses from benign conditions can cause non‐cancer‐related elevations in tumor markers.^9^ Prior research has demonstrated that benign elevations in markers are common^5^, ^10^ and caused by diverse pathologies,^5^, ^10^, ^11^ including pulmonary diseases such as pneumonia,^11^, ^12^ chronic obstructive pulmonary disease,^13^ and pulmonary fibrosis.^14^ The severe acute respiratory coronavirus 2 (SARS‐CoV‐2) induces a distinct viral inflammatory response, resulting in coronavirus disease 2019 (COVID‐19). Clinical data suggest that CEA, CA 19–9, and CA 125 levels might rise in patients with COVID‐19.^15^, ^16^, ^17^ In patients without malignancy, preliminary data show that CEA is elevated above 5.0 ng/ml in approximately one‐fifth (18.7%) to one‐quarter (23.1%) of patients with COVID‐19.^18^, ^19^ Moreover, the magnitude of increase in CEA, CA 19–9, and CA 125 is associated with severity of COVID‐19 infection.^15^, ^17^, ^20^ However, available data excludes cancer patients, so little is known about the effect of COVID‐19 on tumor markers among cancer patients.

Since a rise in markers could be misleadingly associated with cancer progression or recurrence, understanding the effect of COVID‐19 on tumor markers in patients with malignancy is needed to prevent unnecessary treatment changes, diagnostic testing, and increases in patient anxiety. To fill this gap, we present a case series to describe the relationship between COVID‐19 and cancer tumor markers in patients with cancer. Additionally, we briefly synthesize possible mechanisms connecting COVID‐19 infection to serum tumor markers.

## 2 METHODS

After approval from the Duke University Health System institutional review board, we identified patients with a positive SARS‐CoV‐2 PCR test, diagnosis of a solid tumor malignancy, and a CEA, CA 19–9, CA 125, or CA 27–29 laboratory test. Patients were identified using the Duke COVID‐19 registry, which includes clinical information from the electronic health record, as well as through referral from oncology teams at the Duke Cancer Institute. We included cancer patients with at least one pre‐ and two post‐infection tumor marker measurements between January 1, 2020 and August 26, 2021. Patients were excluded if they presented with a sustained elevation of tumor markers in the post‐COVID‐19 period, if a transient increase was present, but not outside the normal reference range, or if no elevation of tumor markers was present in the post‐COVID‐19 period. Additionally, patients were excluded if documented evidence of cancer progression was present. Normal ranges for tumor markers were defined by institutional values, which are CEA <2.5 ng/ml, CA 125 <35 U/ml, CA 19–9 <40 U/ml, and CA 27–29 <38 U/ml.

We abstracted the electronic health record for demographics, cancer diagnosis, treatment, evidence of cancer progression, date and severity of COVID‐19 infection, and tumor marker values. The date of COVID‐19 infection was defined as the date that the patient had documented symptoms of infection. COVID‐19 severity assessed using the World Health Organization (WHO) Clinical Progression Scale.^21^ Radiographic and clinical data in the period between the first and last tumor marker tests was reviewed for evidence of cancer progression and change in cancer treatment.

## 3 RESULTS

**FIGURE 1:**
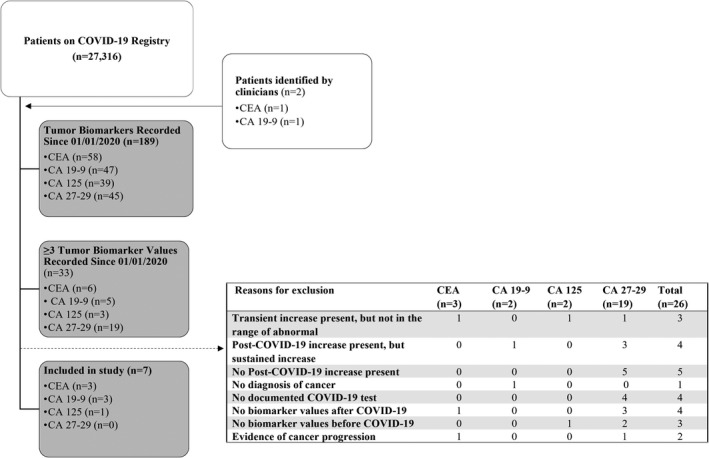
Identification of cancer patients with COIVD‐19 with reasons for exclusion

A total of 27,316 patients were identified on the Duke COVID‐19 registry and two patients were identified by clinicians (Figure 1). Of these, 189 patients had tumor markers recorded during the study period, including 58 patients with CEA values, 47 patients with CA 19–9 values, 39 patients with CA 125 values, and 45 patients with CA 27–29 values. Six patients had at least three CEA values and four were included in the study. Reasons for exclusion were the absence of CEA levels in the post‐COVID‐19 period (*n* = 1), the observed transient increase did not rise above normal range (*n* = 1), and radiographic evidence of cancer progression (*n* = 1). Regarding CA 19–9, five patients had the sufficient number of tumor marker values and three met the inclusion criteria. The excluded patients either displayed a sustained post‐COVID‐19 increase (*n* = 1) or did not have a diagnosis of malignancy (*n* = 1). One of the three patients with at least three CA 125 values were included; reasons for exclusions were insufficient magnitude of transient increase tumor marker (*n* = 1) and the absence of tumor marker measurements from prior to the COVID‐19 infection (*n* = 1). Finally, none out of the 19 patients with at least three CA 27–29 values were included. The most common reasons for exclusion were the absence of a post‐COVID‐19 increase in values (*n* = 5), no documented COVID‐19 test (*n* = 4), sustained post‐COVID‐19 increase (*n* = 3), and the absence of measurements in the post‐COVID‐19 period (*n* = 3).

In total, we identified seven patients with solid tumors and temporary elevation of tumor marker values during the post‐COVID‐19 period. Patient characteristics and cancer tumor marker information are found in Table 1. The diagnoses of included patients were colon cancer (*n* = 1), pancreatic cancer (*n* = 2), gastric cancer (*n* = 2), rectal cancer (*n* = 1), and serous ovarian carcinoma (*n* = 1).With regards to the severity of COVID‐19, four patients were hospitalized with moderate disease (WHO Clinical Progression Scale score of 4 or 5) and the remaining five patients were symptomatic with mild disease (WHO Clinical Progression Scale score of 2). Regarding cancer progression, six patients displayed no evidence of disease progression, while one patient (ID4) did not have any available imaging or other clinical data that evaluated disease progression.

**TABLE 1:** Tumor marker response to COVID‐19 infection in seven patients with cancer

Patient No.	Age, y	Primary cancer diagnosis	Biomarker (units)	Pre‐COVID‐19 (time relation to infection)	Post‐COVID‐19 (time relation to infection)		Severity of COVID‐19 infection (WHO Clinical Progression score)	Evidence of disease progression^a^	Change in cancer treatment^b^
ID1	64	Rectal cancer	CEA (ng/ml)	2.4 (−34 days)	3.3 (56 days)	2.2 (150 days)	Hospitalized moderate disease (5)	No growth or new disease on radiographic study	No change. Continued cancer surveillance without therapy.
ID2	76	Gastric cancer	CEA (ng/ml)	3.8 (−26 days)	9.1 (33 days)	3.9 (179 days)	Ambulatory mild disease (2)	No growth or new disease on radiographic study	No change. Continued cancer surveillance without therapy.
ID3	58	Colon cancer	CEA (ng/ml)	17.3 (−85 days)	23.2 (−2 days)	16.7 (40 days)	Hospitalized moderate disease (5)	No growth or new disease on radiographic study	No change. Continued FOLFOX and bevacizumab.
ID4	65	Ovarian cancer	CA 125 (U/ml)	513.1 (−13 days)	607.3 (−1 day)	419.9 (29 days)	Ambulatory mild disease (2)	Unknown	Change. Therapy withheld in post‐COVID‐19 period.
ID5	72	Gastric cancer	CA 19–9 (U/ml)	259 (−31 days)	436 (20 days)	218 (81 days)	Hospitalized moderate disease (4)	No growth or new disease on radiographic study	Change. Therapy with held in post‐COVID‐19 period.
ID6	63	Pancreatic cancer	CA 19–9 (U/ml)	81 (−40 days)	147 (24 days)	106 (79 days)	Hospitalized moderate disease (4)	No growth or new disease on radiographic study	No change. Continued gemcitabine and paclitaxel.
ID7	55	Pancreatic cancer	CA 19–9 (U/ml)	23 (−50 days)	113 (6 days)	24 (48 days)	Ambulatory mild disease (2)	No growth or new disease on radiographic study	No change. Continued cancer surveillance without therapy.

Abbreviations: FOLFOX, folinic acid, fluorouracil, and oxaliplatin; WHO, World Health Organization.

^a^ Evidence of disease progression documented in clinical note or in radiographic imaging report within the time from the first biomarker to test to the last reported biomarker test.

^b^ Cancer treatment refers to any chemotherapy, radiation, or surgery received during the study period.

**FIGURE 2:**
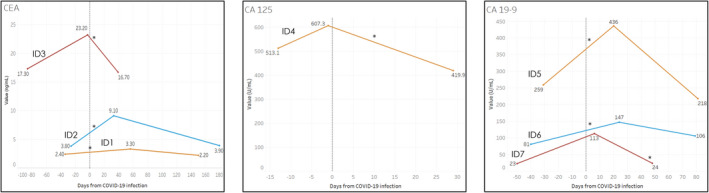
Tumor marker values with relation to COVID‐19 infection. *, refers to date of the positive SARS‐CoV‐2 PCR test with relation to the date of documented COVID‐19 symptoms. CA 19–9, cancer antigen 19–9; CA 125, cancer antigen 125; CEA, carcinoembryonic antigen

Elevation in tumor marker occurred at a median of 20 days after COVID‐19 infection, ranging from 2 days prior to and 56 days following documentation of infection (Figure 2). The magnitude of elevation in tumor marker values was 94.2 for CA 125 and ranged from 0.9 to 19.3 for CEA and 66 to 177 for CA 19–9. Among all patients, tumor markers subsequently decreased at the second time point in the post‐infectious period at a median of 79 days, ranging from 40 to 179 days. Among the included patients, three patients were on follow‐up surveillance, while the remaining six were on active treatment. Two patients had anticancer therapy held during the post‐COVID‐19 infection period.

## 4 DISCUSSION

In this case series, we describe temporary elevations of cancer tumor markers in the period surrounding COVID‐19 infection. To our knowledge this is the first report of this phenomenon in cancer patients and has implications for ongoing cancer surveillance and treatment decisions as well as generating hypotheses for future research. Awareness of falsely elevated tumor markers is important to preventing unnecessary diagnostic testing, alterations in treatment, and patient anxiety. Finally, this work supplements prior research, which identifies a significant increase in tumor markers among non‐cancer patients infected with COVID‐19.^15^, ^16^, ^17^, ^18^, ^19^, ^20^ Despite these clinical observations, the mechanism of benign elevations in tumor markers, like those following COVID‐19 infection, is not known and are hypothesized to stem from the molecular roles of the markers within the pulmonary inflammatory response.^11^, ^14^, ^17^, ^22^

### 4.1 CEA

This case series includes three patients (ID1‐3) with a temporary rise in CEA after contracting COVID‐19 infection. In two of the patients, the pre‐COVID‐19 CEA value was abnormal, likely due to their underlying malignancy. While all three patients displayed the temporary rise in CEA, the timing and intensity differed. For example, in ID3, the observed rise in CEA resolved after 40 days following COVID‐19, while ID1’s first measurement, which demonstrated an elevation, occurred 56 days after infection. The reasons for this are unknown and may be related to disease severity and variability of inflammatory response. In cohort studies of patients without cancer, clinical data shows that up to a quarter of patients with COVID‐19 have elevated CEA values and that the intensity of increase is correlated with severity of COVID‐19.^15^, ^16^, ^17^, ^19^, ^20^ Among patients hospitalized with COVID‐19, the average CEA value was 8.23 ng/ml, which increased to 14.8 ng/ml among those who died and decreased to 3.8 ng/ml among those who were discharged.^17^ Moreover, Chen et al. report serial measurement of CEA in 13 patients while they were hospitalized with COVID‐19 and found that levels began to decrease at a median interval of 24 days following infection.^17^

CEA is a cell surface adhesion molecule and primarily expressed in the gastrointestinal tract, but also noted to occur in the respiratory tract.^23^ Proposed mechanisms for the temporary rise in CEA following COVID‐19 infection include (1) viral immune response,^20^ (2) acute infection‐induced alveolar damage,^17^ and (3) direct gastrointestinal damage.^19^ Prior work has shown that CEA is located in the alveolar epithelial cells and responds to interferon gamma, a key cytokine in the viral immune response.^24^ Additionally, CEA expression occurs in type II pneumocytes,^25^ which may be atypically activated due to COVID‐19‐induced alveolar damage.^26^ Finally, gastrointestinal epithelial cells may be directly affected by the SARS‐CoV‐2 virus, leading to increased CEA levels as the tissue regenerates.^27^

### 4.2 CA 19–9

Three patients (ID5‐ID7) demonstrated a rise in CA 19–9 following COVID‐19 infection. The magnitude of rise differed among the three patients: ID5 increased from 81 U/ml to 147 U/ml, ID6 23 U/ml to 113 U/ml, and ID7 186 U/ml to 281 U/ml. These findings corroborate reports of increased CA 19–9 in patients without cancer who are infected with COVID‐19.^15^ While our findings did not display a relationship between the magnitude of change among CA 19–9 values and the severity of COVID‐19, Wei et al. report that greater increases in CA 19–9 occurred in critical or severe cases compared with mild cases of COVID‐19.^15^

Elevations in CA 19–9 levels from benign pulmonary disease may be common, including in nearly 40% of patients with chronic lung diseases.^28^ CA 19–9 is located in epithelial cells in the gastric and respiratory tracts in addition to the pancreatic parenchyma and biliary tract.^28^, ^29^, ^30^ Prior work suggests that CA 19–9 is elevated in benign lung disease due to the extravasation of mucus hypersecretion from bronchiolar epithelial cells during inflammation.^11^, ^28^, ^31^ Additionally, CA 19–9 levels may be elevated in pulmonary pathologies that block its excretion, such as with bronchitis.^11^ While the precise mechanism for the observed rise during COVID‐19 is unknown, it is likely related to inflammatory processes in the lung.

### 4.3 CA 125

One patient (ID4) was identified with a temporary rise in CA 125 following COVID‐19 infection. This observation supplements reports of CA 125 elevation among patients without cancer who were infected with COVID‐19. Wei et al. reported that patients with mild cases of COVID‐19 had a mean CA 125 of 18.1 U/ml, which was significantly higher than healthy controls (CA 125 10.5 U/ml).^15^ Additionally, Smith et al. reported a case of transient increase in CA 125 in a woman with ovarian serous carcinoma during the COVID‐19 pandemic.^32^ While the patient was not tested with a PCR test at the time of infection, she later had a positive COVID‐19 antibody test, suggesting the rise in CA 125 may be related to the infection.^32^

CA 125 is a glycoprotein synthesized in serous epithelial cells, such as those found in the respiratory tract, ocular surface, and female reproductive tract.^33^ The proposed physiological role of CA 125 is to protect epithelial lumen surfaces from physical stress as the molecule is activated in response to mechanical and oxidative stress as well as inflammatory cytokines.^22^, ^34^, ^35^ Prior work has shown that CA 125 is elevated in patients with benign lung pathologies, such as pneumonia, and are associated with pleural effusions.^22^, ^36^ CA 125’s role in responding to stress is a potential mechanism for the COVID‐19 infection to cause transient elevations in the tumor marker.

### 4.4 CA 27–29

Of the 19 patients with at least three recorded CA 27–29 values, none met the inclusion criteria for this study. This absence supports prior clinical studies in non‐cancer patients, which either did not examine CA 27–29 or did not report a transient elevation.^15^, ^16^, ^17^, ^18^, ^19^, ^20^Although an association of COVID‐19 and CA 27–29 levels has not been observed, there are prior published reports of elevated CA 27–29 in benign pulmonary disease. Kurian et al. report a series of patients with chronic lung diseases and persistently elevated CA 27–29.^14^ However, no data have supported a link between CA 27–29 and COVID‐19.

### 4.5 Limitations

The primary limitations of this study are the result of a small sample size. We were limited in our identification of cancer patients for multiple reasons, including patients' decline in healthcare utilization while isolating with COVID‐19 and the high mortality of COVID‐19 among cancer patients. Additionally, we cannot rule out that the observed temporary elevations are the result of laboratory errors or due to dynamics of underlying carcinogenesis. Despite these limitations, this case series has key strengths, including the presentation of novel observations regarding the transient rise in serum tumor markers in patients with cancer after contracting COVID‐19. Future work should examine this process in larger, prospective samples to confirm the results from this study and to assess the effect of COVID‐19 severity on the relative change in value and length of sustained response.

## 5 CONCLUSION

In conclusion, we found temporary elevations of cancer tumor markers in the period surrounding COVID‐19 infection in seven patients. Our work suggests that confirmation of tumor marker elevation prior to altering treatment strategies or pursuing radiographic testing may be appropriate in cancer patients with COVID‐19. Moreover, this work can be used as the basis of future research in this area to better understand the effect of COVID‐19 on serum tumor markers.

## CONFLICT OF INTEREST

None.

## AUTHOR CONTRIBUTIONS

**Alexander Gunn:** Conceptualization, methodology, data analysis and interpretation, writing‐ original draft, and writing‐ review and editing. **Carolyn Tashie:** Conceptualization, and writing‐ review and editing. **Steven Wolf:** Methodology, data interpretation, and writing‐review and editing. **Jesse Troy:** Methodology, data interpretation, and writing‐review and editing. **S. Yousuf Zafar:** Conceptualization, supervision, methodology, data analysis and interpretation, writing‐ original draft, and writing‐ review and editing.

## ETHICS APPROVAL STATEMENT

This research was reviewed and approved by the Duke University Health System Institutional Review Board prior to the start of the study.

## Data Availability

The data that support the findings of this study are available from the corresponding author upon reasonable request.

## References

[1] Network NCC . Colon cancer (version 32021). 2021. Accessed September 15, 2021. https://www.nccn.org/professionals/physician_gls/pdf/colon.pdf

[2] Network NCC . Pancreatic adenocarcinoma (version: 2.2021). 2021. Accessed September 15, 2021. https://www.nccn.org/professionals/physician_gls/pdf/pancreatic.pdf

[3] Network NCC . Breast cancer (version 8.2021). 2021. Accessed September 15, 2021. https://www.nccn.org/professionals/physician_gls/pdf/breast.pdf

[4] Network NCC . Ovarian cancer/fallopian tube cancer/primary peritoneal cancer (version: 3.2021). 2021. Accessed September 15, 2021. https://www.nccn.org/professionals/physician_gls/pdf/ovarian.pdf 10.6004/jnccn.2024.0052 39413835

[5] Litvak A , Cercek A , Segal N , et al. False‐positive elevations of carcinoembryonic antigen in patients with a history of resected colorectal cancer. J Natl Compr Cancer Netw. 2014;12(6):907‐913. doi:10.6004/jnccn.2014.0085 PMC8409326 24925201

[6] Dahele M , Camidge R . Tumour marker reference ranges and patients' anxiety. Lancet. 2003;361(9360):882. doi:10.1016/s0140-6736(03)12697-9 12642085

[7] Reid A , Ercolano E , Schwartz P , McCorkle R . The Management of Anxiety and Knowledge of serum CA‐125 after an ovarian cancer diagnosis. Clin J Oncol Nurs. 2011;15(6):625‐632. doi:10.1188/11.cjon.625-632 22119973

[8] Huang X , Zhang TZ , Li GH , Liu L , Xu GQ . Prevalence and correlation of anxiety and depression on the prognosis of postoperative non‐small‐cell lung cancer patients in North China. Medicine (Baltimore). 2020;99(11):e19087. doi:10.1097/md.0000000000019087 32176035 PMC7440182

[9] Perkins GL , Slater ED , Sanders GK , Prichard JG . Serum tumor markers. Am Fam Physician. 2003;68(6):1075‐1082. 14524394

[10] Moss EL . The role of CA125 in clinical practice. J Clin Pathol. 2005;58(3):308‐312. doi:10.1136/jcp.2004.018077 15735166 PMC1770590

[11] Kim S , Park BK , Seo JH , et al. Carbohydrate antigen 19‐9 elevation without evidence of malignant or pancreatobiliary diseases. Sci Rep. 2020;10(1):8820. doi:10.1038/s41598-020-65720-8 32483216 PMC7264353

[12] Stockley RA , Shaw J , Whitfield AG , Whitehead TP , Clarke CA , Burnett D . Effect of cigarette smoking, pulmonary inflammation, and lung disease on concentrations of carcinoembryonic antigen in serum and secretions. Thorax. 1986;41(1):17‐24. doi:10.1136/thx.41.1.17 3704962 PMC460246

[13] Li S , Ma H , Gan L , et al. Cancer antigen‐125 levels correlate with pleural effusions and COPD‐related complications in people living at high altitude. Medicine. 2018;97(46):e12993. doi:10.1097/md.0000000000012993 30431573 PMC6257551

[14] Kurian S , Khan M , Grant M . CA 27‐29 in patients with breast cancer with pulmonary fibrosis. Clin Breast Cancer. 2008;8(6):538‐540. doi:10.3816/CBC.2008.n.067 19073511

[15] Wei X , Su J , Yang K , et al. Elevations of serum cancer biomarkers correlate with severity of COVID‐19. J Med Virol. 2020;92(10):2036‐2041. doi:10.1002/jmv.25957 32347972 PMC7267262

[16] Yu J , Nie L , Wu D , et al. Prognostic value of a clinical biochemistry‐based nomogram for coronavirus disease 2019. Front Med (Lausanne). 2020;7:597791. doi:10.3389/fmed.2020.597791 33537326 PMC7848223

[17] Chen Q , Kong H , Qi X , et al. Carcinoembryonic antigen: a potential biomarker to evaluate the severity and prognosis of COVID‐19. Front Med (Lausanne). 2020;7:579543. doi:10.3389/fmed.2020.579543 33123542 PMC7573292

[18] Yu J , Yang Z , Zhou X , et al. Prognostic value of carcinoembryonic antigen on outcome in patients with coronavirus disease 2019. J Infect. 2020;81(2):e170‐e172. doi:10.1016/j.jinf.2020.06.018 32540459 PMC7290182

[19] Yang C , Wang J , Liu J , Huang S , Xiong B . Elevated carcinoembryonic antigen in patients with COVID‐19 pneumonia. J Cancer Res Clin Oncol. 2020;146(12):3385‐3388. doi:10.1007/s00432-020-03350-3 32857179 PMC7453671

[20] Huang W , Li M , Luo G , et al. The inflammatory factors associated with disease severity to predict COVID‐19 progression. J Immunol. 2021;206(7):1597‐1608. doi:10.4049/jimmunol.2001327 33579725

[21] Marshall JC , Murthy S , Diaz J , et al. A minimal common outcome measure set for COVID‐19 clinical research. Lancet Infect Dis. 2020;20(8):e192‐e197. doi:10.1016/s1473-3099(20)30483-7 32539990 PMC7292605

[22] Howe T , Sokolovsky N , Sayasneh A , Omar K , Tahmasebi F . Raised CA125—what we actually know…. Obstet Gynaecol. 2021;23(1):21‐27. doi:10.1111/tog.12704

[23] Hammarström S . The carcinoembryonic antigen (CEA) family: structures, suggested functions and expression in normal and malignant tissues. Semin Cancer Biol. 1999;9(2):67‐81. doi:10.1006/scbi.1998.0119 10202129

[24] Klaile E , Klassert TE , Scheffrahn I , et al. Carcinoembryonic antigen (CEA)‐related cell adhesion molecules are co‐expressed in the human lung and their expression can be modulated in bronchial epithelial cells by non‐typable Haemophilus influenzae, Moraxella catarrhalis, TLR3, and type I and II interferons. Respir Res. 2013;14(1):85. doi:10.1186/1465-9921-14-85 23941132 PMC3765474

[25] Noguchi T , Yamamoto K , Moriyama G , et al. Evaluation of serum levels of carcinoembryonic antigen in allergic bronchopulmonary aspergillosis. J Nippon Med Sch. 2013;80(6):404‐409. doi:10.1272/jnms.80.404 24419710

[26] Fox SE , Akmatbekov A , Harbert JL , Li G , Quincy Brown J , Vander Heide RS . Pulmonary and cardiac pathology in African American patients with COVID‐19: an autopsy series from New Orleans. Lancet Respir Med. 2020;8(7):681‐686. doi:10.1016/s2213-2600(20)30243-5 32473124 PMC7255143

[27] Yang L , Tu L . Implications of gastrointestinal manifestations of COVID‐19. Lancet Gastroenterol Hepatol. 2020;5(7):629‐630. doi:10.1016/s2468-1253(20)30132-1 32405602 PMC7217632

[28] Kodama T , Satoh H , Ishikawa H , Ohtsuka M . Serum levels of CA19‐9 in patients with nonmalignant respiratory diseases. J Clin Lab Anal. 2007;21(2):103‐106. doi:10.1002/jcla.20136 17385665 PMC6648978

[29] Shimizu Y , Hamada T , Tanaka Y , Sasaki A , Nemoto T . Colocalization of CA19‐9 and KL‐6 to epithelial cells in dilated bronchioles in a patient with idiopathic pulmonary fibrosis complicated by diffuse alveolar damage. Respirology. 2002;7(3):281‐284. doi:10.1046/j.1440-1843.2002.00391.x 12153695

[30] Galli C , Basso D , Plebani M . CA 19‐9: handle with care. Clin Chem Lab Med. 2013;51(7):1369‐1383. doi:10.1515/cclm-2012-0744 23370912

[31] Kim HR , Lee CH , Kim YW , Han SK , Shim YS , Yim JJ . Increased CA 19‐9 level in patients without malignant disease. Clin Chem Lab Med. 2009;47(6):750‐754. doi:10.1515/cclm.2009.152 19402792

[32] Smith M , Lara OD , Pothuri B . Transient rise in CA 125 in a woman with ovarian carcinoma and COVID‐19 infection. Gynecol Oncol Rep. 2020;34:100644. doi:10.1016/j.gore.2020.100644 32964093 PMC7497776

[33] Bischof P . What do we know about the origin of CA 125? Eur J Obstet Gynecol Reprod Biol. 1993;49(1–2):93‐98. doi:10.1016/0028-2243(93)90131-u 8365529

[34] Núñez J , Miñana G , Núñez E , Chorro FJ , Bodí V , Sanchis J . Clinical utility of antigen carbohydrate 125 in heart failure. Heart Fail Rev. 2014;19(5):575‐584. doi:10.1007/s10741-013-9402-y 23925386

[35] Hung C‐L , Hung T‐C , Lai Y‐H , Lu C‐S , Wu Y‐J , Yeh H‐I . Beyond malignancy: the role of carbohydrate antigen 125 in heart failure. Biomark Res. 2013;1(1):25. doi:10.1186/2050-7771-1-25 24252645 PMC4177553

[36] Topalak O , Saygili U , Soyturk M , et al. Serum, pleural effusion, and ascites CA‐125 levels in ovarian cancer and nonovarian benign and malignant diseases: a comparative study. Gynecol Oncol. 2002;85(1):108‐113. doi:10.1006/gyno.2001.6575 11925128

